# Effects of picosecond laser on the multi-colored tattoo removal using Hartley guinea pig: A preliminary study

**DOI:** 10.1371/journal.pone.0203370

**Published:** 2018-09-06

**Authors:** Mi Soo Choi, Hee Seok Seo, Jong Gu Kim, Sung Jay Choe, Byung Cheol Park, Myung Hwa Kim, Seung Phil Hong

**Affiliations:** 1 Department of Dermatology, College of Medicine, Dankook University, Cheonan, Chungnam, Republic of Korea; 2 Wellness skin research center, Cheonan, Chungnam, Republic of Korea; 3 Cheonan Oracle Dermatology Clinic, Cheonan, Chungnam, Republic of Korea; 4 Department of Dermatology, Yonsei University Wonju College of Medicine, Wonju, Gangwon, Korea; University of Vigo, SPAIN

## Abstract

Picosecond lasers have emerged as the leading technology for tattoo removal due to their shorter pulse lengths. To clarify the features of picosecond lasers, we compared picosecond and nanosecond lasers in their ability to remove multi-colored tattoo in an animal model. We first compared a nanosecond quality-switched Nd:YAG laser with picosecond Alexandrite and quality-switched Nd:YAG lasers and then the picosecond quality-switched Nd:YAG laser with the picosecond Alexandrite laser, using a guinea pig model. The colors in the tattoos included red, orange, yellow, green, blue, and black. Guinea pigs were treated for one session with each type of laser. The clearance of pigmentation and local reactions were evaluated based on clinical photographic assessment, quantitative assessment using a colorimeter, histopathology, and electron microscopic examination before laser treatment, immediately after, and at 3 weeks after the treatment. Regardless of pulse duration, a 532-nm laser was the most effective in clearing red, orange, and yellow pigments, although the overall effect and safety was better with the picosecond 532 nm laser. A picosecond 755 nm laser demonstrated excellent efficacy in removing only green and blue pigments. a picosecond 1064 nm laser demonstrated some effects on non-black colored tattoos. In terms of safety, picosecond lasers produced less tissue injury than nanosecond lasers. Conclusively, picosecond lasers are more effective and safer than nanosecond lasers.

## Introduction

Tattoos have long been used to express an artistic perspective on the body. Like the Western countries, tattooing in Korea has been gaining popularity [1]. The most common color used for tattoos is black; however, colorful tattoos that use multiple dyes have become increasingly popular [2]. With the increasing popularity of tattoos, the demand for an effective method for tattoo removal, such as lasers, has also increased; however, tattoo removal has become complicated due to the use of multiple colors in modern tattoos.

For decades, quality-switched (QS) lasers capable of delivering short pulses of laser energy in the nanosecond domain have been the optimal devices for selective removal of tattoos from the skin. Although the exact mechanism of laser tattoo removal is not completely understood, the theory of selective photothermolysis (SPTL) is believed to be the primary principle of laser tattoo removal. The pigments in a tattoo can be destroyed with minimal or no damage to the surrounding tissues by delivering a sufficient amount of energy to the target area in pulse durations that are equal to or less than the thermal relaxation time (TRT) of the target tissue. TRT is defined as the time required for the heated chromophore to dissipate half the absorbed heat into the surrounding tissues [3]. Therefore, a shorter pulse length accelerates the disruption of the target chromophore by the laser beam while also reducing the risk of thermal injury to the surrounding tissues. These disrupted small particles are then easily removed by the host phagocytes and the lymphatic system.

Therefore, pulse durations that are shorter than the target area’s TRT are important in cosmetic laser treatments of pigmentation therapy. However, the range of nanosecond-domain pulse duration is still too long to completely break the tattoo ink into smaller particles. Most tattoo pigments have a TRT in the range of picoseconds since the size of tattoo particles ranges between 40–300 nm in vivo [4, 5]. The TRTs for carbon particles of 40, 100, 200, and 300 nm diameters are 19.12, 119.5, 478, and 1,060 ps, respectively, according to the TRT formula [6]. Therefore, picosecond lasers with pulse lengths that most closely match the TRT of the tattoo pigment molecules have been recently introduced to improve the efficacy of tattoo removal. These picosecond pulses are expected to allow for the most effective delivery of thermal radiation to tattoo particles through more specific photothermal and photomechanical effects than do traditional nanosecond-domain lasers. They also have an additional effect of minimizing the collateral damage to the surrounding dermis and epidermis [4,6].

Based on this theoretical background, we conducted a pre-clinical study using animal tattoo models to compare the efficacy and safety of picosecond-domain QS Nd:YAG and Alexandrite lasers with that of a conventional, nanosecond-domain QS Nd:YAG laser. Additionally, two representative picosecond laser modules (Alexandrite vs. Nd:YAG lasers) were compared.

## Materials and methods

### Subjects and colored tattooing

Hartley guinea pigs (Japan SLC, Japan) weighing 600–700 g each were used because their backs have a large area and thickness that is similar to that of the human skin. The guinea pigs were fed commercial chow and distilled water and housed individually in polycarbonate cages that were maintained at a constant temperature (22 ± 1°C) and humidity (60%). This study was carried out in strict accordance with the recommendations in the Guide for the Care and Use of Laboratory Animals of the National Institutes of Health. The protocol was approved by the ethics committee of Dankook University (DKU-17-007). We purchased commercially available tattoo inks (Intenze, United Kingdom). The ingredients of the six colored tattoo inks (red, orange, yellow, green, blue, and black) are listed in S1 Table. The backs of the animals were shaved with an electric razor, and they were anaesthetized with intravenous administration of approximately 350 ul per 500–600 g of medetomidine hydrochloride diluted with normal saline in a ratio of 1:3. Tattooing was performed with the six inks using a tattoo pen with reciprocating vibrator-driven needles to a depth of approximately 1 mm with multiple passes within the same square area. Six colored squares were placed on the back of each animal (n = 2 in each treatment group, a total of 12 guinea pigs were used). Each tattoo square measured approximately 1.2 x 1.2 cm. Four weeks after tattooing, the guinea pigs were anesthetized, shaved again as described above, and treated with each laser setting. A 10% xylocaine pump spray was used at the treatment site to reduce the pain before tattooing and laser treatment.

### Laser treatment

To evaluate the effects of picosecond lasers on multi-colored tattoos and compare the characteristics of each laser, we used two picosecond lasers [Picosure^®^ (Cynosure, Massachusetts, USA) and Picoway^®^ (Syneron Candela, California, USA)] and one conventional QS Nd:YAG laser [HELIOS^®^, Laseroptek, Seongnam, Korea]. The following laser treatment sets (wavelength/pulse duration/spot size/fluence) were used: 532 nm/10 ns/3 mm/2.3 Jcm^-2^ and 1064 nm/10 ns/3 mm/3.4 Jcm^-2^ for Nd:YAG; and 532 nm/375 ps/5 mm/0.8 Jcm^-2^, 755 nm/750 ps/2.8 mm/3.3 Jcm^-2^, and 1064 nm/450 ps/3 mm/3 Jcm^-2^ for picosecond lasers. The treatments were performed in a single session. The 755-nm QS Alexandrite laser was not included in the experiment because it is rarely used today and therefore was not available.

### Clinical assessments

#### 1. Clinical photographic assessment

Clinical photographic assessment was performed by four dermatologists blinded to the study, based on a standard digital photograph taken by a digital camera (Canon 700D, Tokyo, Japan) under similar conditions (light source, room, and camera) at baseline, before treatment, immediately after treatment, and at 3 weeks after the treatment. To assess the efficacy of laser treatment, the tattoo removal rate was scored on a five-point (0–4) scale by each of four investigators as follows: 4: ≥ 70% removal, excellent improvement; 3: ≥ 50% and < 70%, marked improvement; 2: ≥ 30% and < 50%, moderate improvement; 1: ≥ 10% and < 30%, mild improvement; and 0: < 10%, no effect. Subsequently, each evaluated score was averaged.

Results are expressed as means and standard deviations (SD) of means. SPSS for windows (version 20, IBM, New York, USA) was used for Student’s t-test. Data with 95% confidence intervals were obtained. A p-value of less than 0.05 (p<0.05) was considered statistically significant.

#### 2. Quantitative assessment using a colorimeter

Each tattoo color was objectively measured at two spots within the tattoo area using a colorimeter (CR-400, Minolta, Tokyo, Japan) at baseline and 3 weeks after the treatment. The mean of the two values was presented. L* values represent the white (-) to black (+) color changes in the tattoo; a* values represent the red (-) to green (+) color changes; and b* values represent the yellow (-) to blue (+) color changes. To assess the changes in pigmentation, L* values alone were used to evaluate the black areas of the tattoos, a* values the red and green areas, and b* values the yellow and blue areas. The change in pigmentation from baseline was calculated using the following formula:

$$\%\;\mathrm{change}\;\mathrm{in}\;\mathrm{pigmentation}=\frac{post-treatment\;(at\;3\;weeks)\;colorimetric\;result-baseline\;colorimetric\;result}{baseline\;colorimetric\;result}\;\times 100$$

To assess the efficacy of laser treatment, we divided the changes in pigmentation into the 5 groups mentioned previously.

#### 3. Ultrastructural evaluation by electron microscopy (EM)

Biopsy samples were taken immediately after laser treatment and fixed by modified Karnovsky’s fixation at 4°C for 24 hours. These samples were then rinsed with 0.1 M sodium cacodylate buffer and post-fixed with 1% osmium tetroxide in the same buffer for 2 hours. After rinsing with 0.1 M cacodylate buffer, they were dehydrated for 15 minutes in increasing concentrations of ethanol (70, 80, 90, 95, and 100% v/v), exchanged through propylene oxide, and embedded in a mixture of epoxy resin. Sections were cut using ULTRACUT E ultra-microtome (Reichert-Jung, Austria) and were stained with 1% uranyl acetate for 14 minutes, followed by a lead staining reagent for 3 minutes. The sections were examined with a transmission electron microscope JEM 1200 EXⅡ (JEOL, Japan).

#### 4. Histopathologic evaluation

Full thickness skin samples were obtained using a 4-mm punch biopsy of each colored tattoo area and stained with hematoxylin and eosin (H&E). Under light microscopy, the proportion of tattoo pigment area per unit area of H&E was measured twice and the average values were compared for all the control and experimental skin sites. To quantify the proportion of stained area per unit area, photographs were blindly taken over a constant area of each skin sample. The photographs were then used by evaluators blinded to the study to assess the proportion of stained area using the software Photoshop (Adobe Inc., San Jose, CA, USA).

## Results

### Clinical photographic assessments

**Fig 1:**
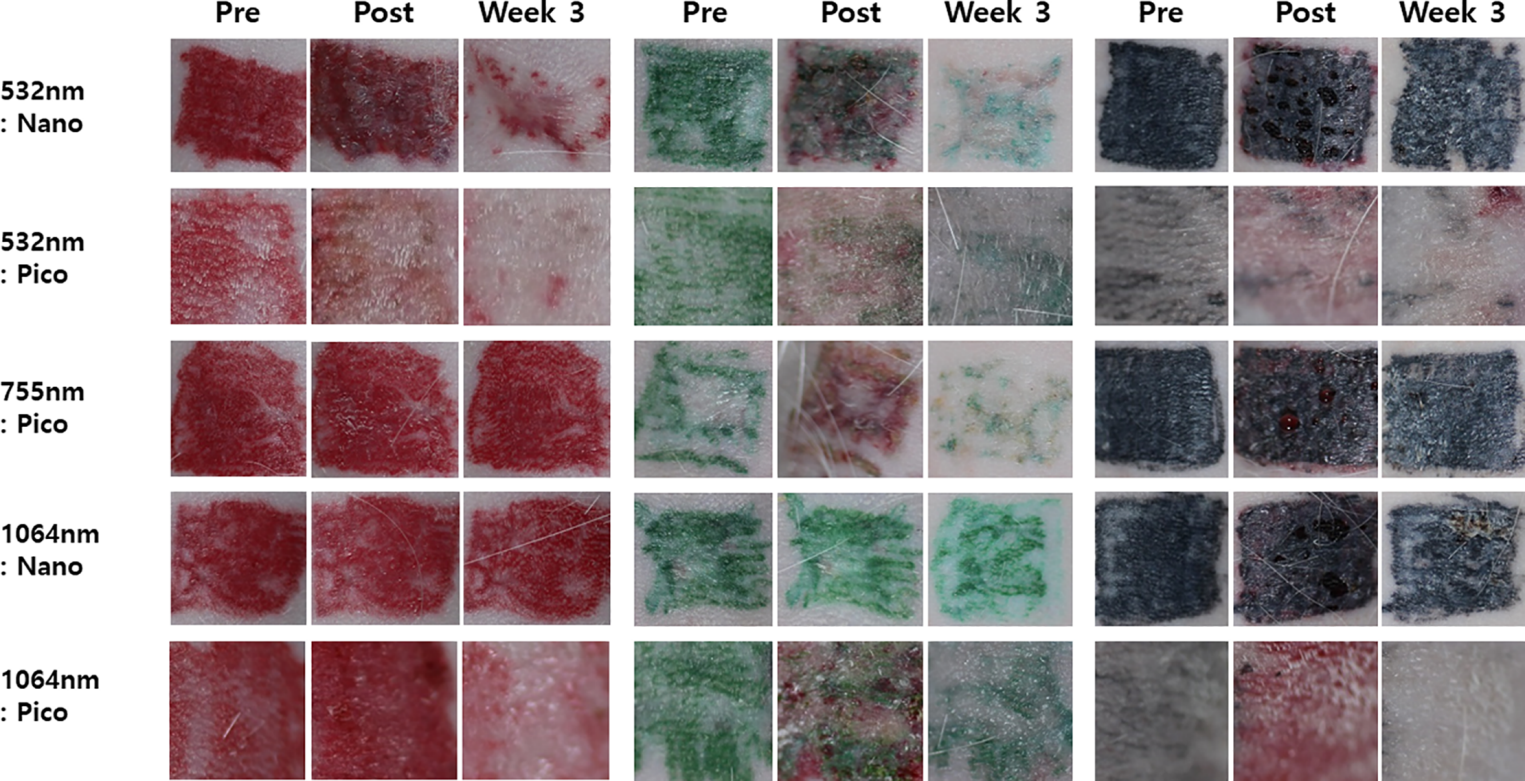
Clinical photographs of removal of red, green, and black pigments over time. The 532 nm lasers were the most effective in removing red colored tattoos by week 3. The 532 nm picosecond laser resulted in less epidermal damage post-treatment and rapid healing than the 532-nm nanosecond laser. The 755 nm picosecond laser was the most effective in removing green colored tattoos. Note: Pre: pre-treatment; Post: immediately after laser treatment; Week 3: 3 weeks after the laser treatment.

Regardless of the pulse duration of the laser, 532 nm wavelength lasers were superior to other wavelength lasers of 755 and 1064 nm in removing warm colored (red, orange, and yellow) tattoos (Fig 1, S1 Fig, Table 1). The 755 nm picosecond laser and 1064 nm nanosecond laser had minimal effects in removing these colors. Interestingly, unlike the 1064 nm nanosecond laser, the 1064 nm picosecond laser showed marked efficacy in removing red and mild efficacy in removing orange and yellow colored tattoos.

**Table 1:** The mean scores of tattoo removal rate on clinical photographic assessment.

Treatment modality		Red	Orange	Yellow	Green	Blue	Black
Wavelength	Pulse duration						
**532**	**nanosec.**	3.75 (±0.5)	3.25 (±0.5)	3.75 (±0.5)	3.00 (±0.0)	1.25 (±0.5)	0.75 (±0.5)
**532**	**picosec.**	4.00 (±0.0)	4.00* (±0.0)	4.00 (±0.0)	2.75 (±0.5)	0.75 (±0.5)	2.75** (±0.5)
**755**	**picosec.**	0.00^##^^,^^$ $^ (±0.0)	0.00^##^^,^^$ $^ (±0.0)	0.25^##^^,^^$^ (±0.5)	3.50^$ $^ (±0.58)	3.75^##^^,^^$ $^ (±0.5)	0.75^##^^,^^$ $^ (±0.5)
**1064**	**nanosec.**	0.50**^,^ ^##^ (±0.58)	0.00**^,^ ^##^ (±0.0)	0.50**^,^^##^ (±0.58)	1.00**^,^^##^ (±0.0)	0.00** (±0.0)	1.50^##^ (±0.58)
**1064**	**picosec.**	3.25^##^^,^^&&^ (±0.5)	1.25^##^^,^^&&^ (±0.5)	1.50^##^^,^^&^ (±0.58)	2.00^#^^,^^&&^ (±0.0)	0.50 (±0.58)	4.00^##^^,^^&&^ (±0.0)

The values are presented as mean (± standard deviation)

*: p< 0.05

**: p<0.01; vs 532 nm nanosec.

#: p< 0.05

##: p<0.01; vs 532 nm picosec.

&: p< 0.05

&&: p< 0.05; vs 1064 nm nanosec.

$: p< 0.05

$ $: p< 0.05; vs 1064 nm picosec.

For blue and green colored tattoo removal, the 755 nm picosecond laser was the most effective (Fig 1, S1 Fig, Table 1). The 532 nm lasers with both nanosecond and picosecond pulse durations were also effective in removing green color but had little effect on blue color. The 1064 nm nanosecond laser showed minimal efficacy in green and blue colored tattoo removal, while the 1064 nm picosecond laser was moderately effective in removing green colored tattoos. For removing black colored tattoos, 532 nm picosecond laser was moderately effective and 1064 nm picosecond laser showed excellent efficacy over other lasers.

In terms of overlying epidermal damage, the 532 nm nanosecond laser-treated lesions demonstrated the most damaged appearance immediately after the treatment including hemorrhage and loss of epidermal tissue, while the 532 nm picosecond laser-treated lesions also demonstrated fewer epidermal changes compared with those treated by the 532 nm nanosecond laser (Fig 1). Overall, none of the laser treatments caused serious skin damage immediately after the treatment, and the 1064 nm nanosecond laser resulted in the least epidermal damage.

### Quantitative assessment using colorimeter

**Fig 2:**
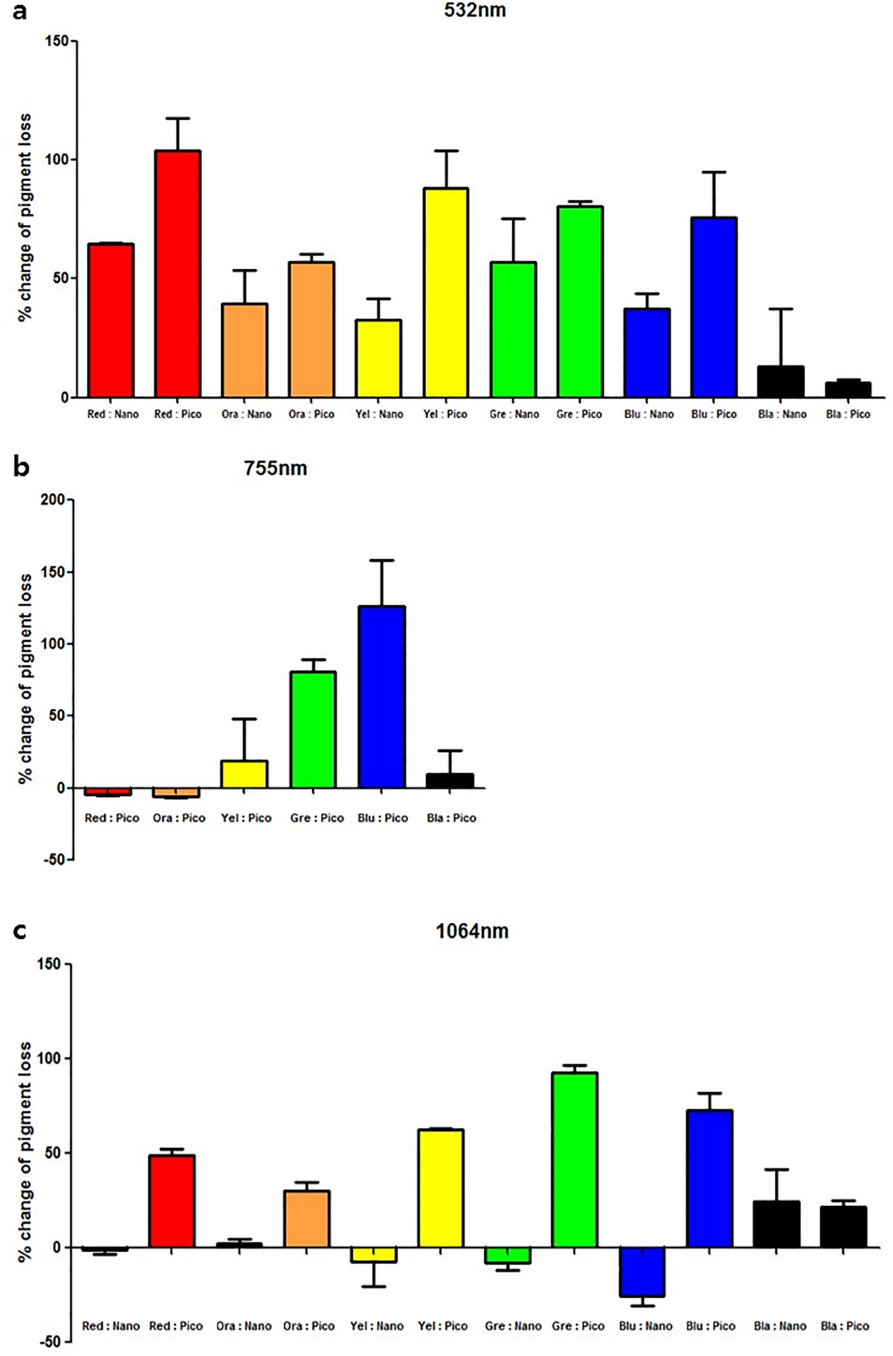
Colorimetric analysis. (a) Colorimetric intensity was measured and compared before and 3 weeks after the treatment. The 532 nm picosecond laser exhibited > 50% change in pigment intensity in all colors except black. (b) The 755 nm picosecond laser was only effective in removing green and blue colors, and the changes in green and blue pigment intensities were the greatest. (c) The 1064 nm nanosecond laser had almost no effect in removing any color except black. However, the 1064-nm picosecond laser resulted in > 50% decrease in yellow, green, and blue pigments, while also effectively reducing red and orange colors.

The efficacies of picosecond and nanosecond laser therapies for each color were compared using colorimetric measurements. At 532 nm wavelength, the picosecond laser demonstrated excellent improvements in all colors of tattoos except black and an overall superior efficacy compared with the nanosecond laser; however, both lasers showed relatively less changes and similar efficacies in removing black colored tattoos (Fig 2A).

At 755 nm wavelength, the nanosecond laser was unavailable; therefore, we only evaluated the results of the picosecond laser. The 755 nm picosecond laser was found to be excellent in treating green and blue colored tattoos but its efficacy was limited to these two colors (Fig 2B).

The most obvious difference between picosecond and nanosecond laser was at 1064 nm wavelength. The 1064-nm picosecond laser demonstrated excellent efficacy in removing blue (% change of pigmentation: 73.08%) and green (92.60%), marked efficacy in removing yellow (62.40%), and moderate efficacy in removing red (49.04%) and orange (30.32%), and mild efficacy in removing black colored tattoos (21.57%) (Fig 2C). Although the 1064-nm nanosecond laser had minimal effect in removing colored tattoos, its effects were similar to those of the picosecond laser in removing black colored tattoos.

### Ultrastructural evaluation by electron microscopy (EM)

**Fig 3:**
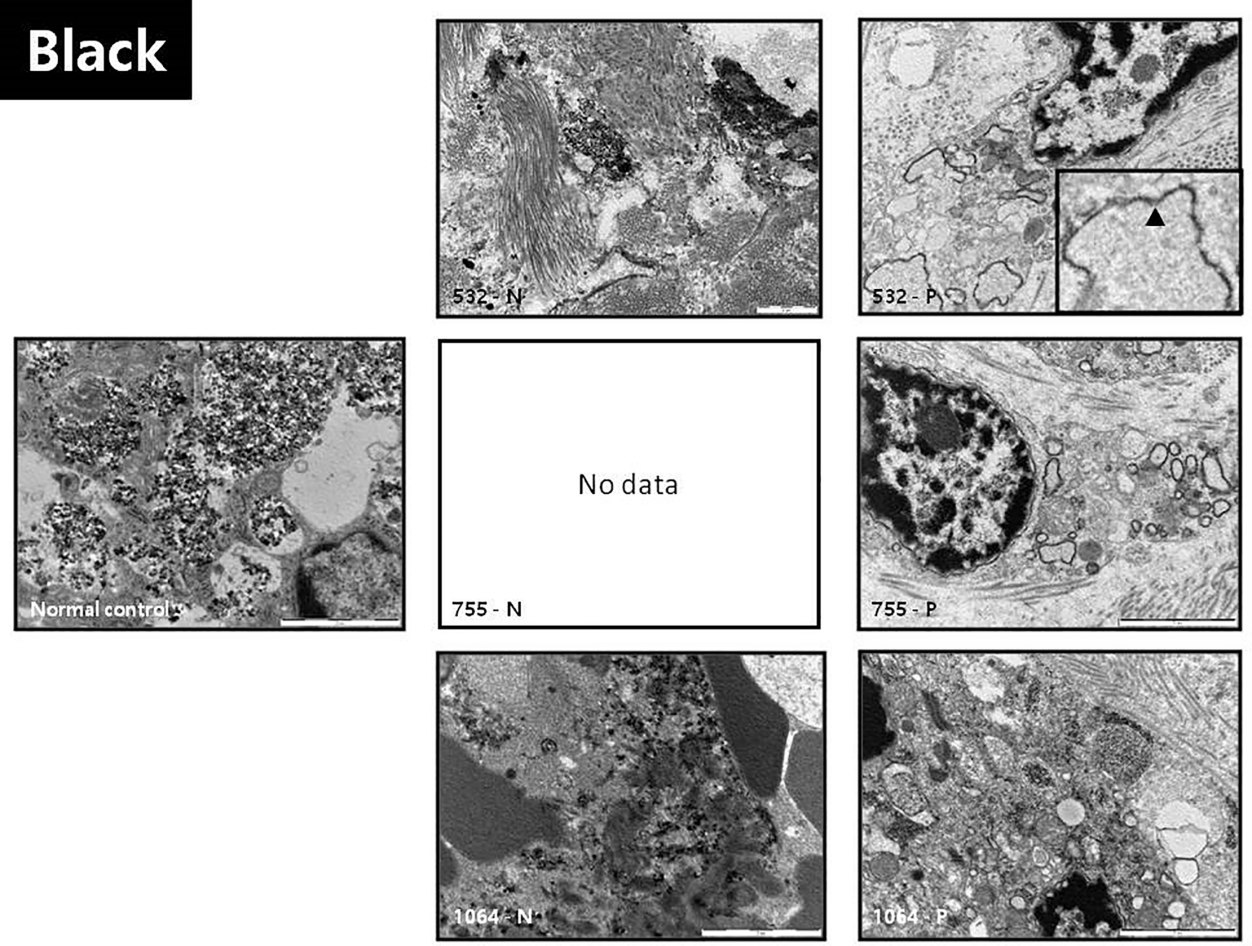
Ultrastructural analysis of colored tattoos using electron microscopy (EM). The EM results following treatment with picosecond lasers in all colors revealed a typical rimming pattern along the peripheries of the lysosomal lobules. Unlike the picosecond laser, the nanosecond laser treatment resulted in tattoo particles that left the lysosomal structure and were scattered in cellular spaces, thereby disrupting the cell structures (inlet: a magnified photo of the rimming pattern; black arrowhead: rimming pattern of tattoo particles) (bar = 2 μm).

To investigate the differences in the mechanism of action of picosecond and nanosecond lasers by observing microstructure changes, transmission electron microscopy (TEM) studies were conducted in samples obtained before (normal control) and immediately after the treatment. We defined “particle” as the smallest identifiable pigment structure on EM. First, coarse electron dense particles of black tattoo pigment were compactly aggregated together to form multiple lysosomal lobules within a macrophage cytoplasm within the dermis of the normal control animals (Fig 3, normal control). Immediately after 532 nm nanosecond laser treatment, the aggregated lobular structures disappeared and only some electron dense particles were scattered in the extracellular space between the dermal collagen bundles outside the cellular structure (Fig 3, 532-N). In 1064 nm nanosecond laser treated dermis, more particles were observed and the effect of the laser was less noticeable (Fig 3, 1064-N).

Instead of scattered electron-dense particles in the dermal interspace, or the cytoplasmic lobules containing the remaining particles after nanosecond laser-treatment, some vesicular structures with an empty center and characteristic electron dense annular rim were observed after treatment with the picosecond laser. Furthermore, the electron-dense particles were seldom found inside the cells. Nevertheless, the cell contours and cellular membranes were relatively well-maintained. Since the annular vesicular structure was clearly observed in other colors samples that were treated with picosecond lasers, this characteristic annular structure was believed to be associated with pulse duration of picosecond lasers.

The blue tattoo pigments also exhibited aggregated lysosomal lobular structures similar to those exhibited by the black tattoo pigment; however, the particles within the lobule were finer (S2 Fig, normal control). After 532 nm and 755 nm picosecond laser treatments, fine electro-dense particles within the lysosomal lobules were broken into smaller ones and demonstrated the characteristic rimming pattern of the picosecond laser (S2 Fig).

The normal ultrastructure of red color tattoo exhibited mixed, slit-like electron-lucent materials inside the cellular space, thereby forming electron-less bright lysosomal lobules (S3 Fig). After treatment with the 532 nm nanosecond laser, the electron-lucent lobules of the red colored tattoo were disrupted and rod-like materials escaped into the cellular space outside the lysosome, resulting in a pattern similar to that of black tattoo. After treatment with the 532 nm picosecond laser, rod-like materials were not nearly seen and the typical rimming pattern of picosecond lasers was found in the picosecond laser-treated lesions (S3 Fig).

Tattoo particles from green, yellow, and orange colored tattoo pigments demonstrated some empty round space and electron-dense round material inside the electron-lucent lobules with similar EM findings between them (S4, S5 and S6 Figs). The characteristic rimming pattern of fine particles was also observed after picosecond laser treatment, which was similar to the outcomes in treatments of black and blue colored tattoos.

### Histopathologic analysis

To compare the post-treatment histological changes, blue and red colored samples was obtained immediately after each laser treatment (S7 Fig). In the 532 nm nanosecond laser-treated sample, large vacuoles and massive red blood cell (RBC) extravasation in the dermal interstitium as well as basal vacuolar changes in the epidermis were observed. In contrast, the 755 nm picosecond laser resulted in the least epidermal and dermal damages (Table 2).

**Table 2:** Summary of histopathologic findings on tissue damage.

Post-treatment damage	532 nm nanosecond	1064 nm nanosecond	755 nm picosecond
**RBC extravasation**	**+++**	**+++**	**++**
**Vacuolar alteration**	**+++**	**++**	**++**
**DEJ cleft**	**+++**	**+**	**+**
**Subcorneal separation**	**++**	**+++**	**+++**

0 : none; + : minimal; ++ : moderate; +++ : severe

## Discussion

To further clarify the features of picosecond lasers, we compared the differences in the effects according to the wavelength and pulse duration using a multi-colored tattoo animal model. The results are summarized in Table 3. The Nd:YAG laser was used at 532 and 1064 nm, and the Alexandrite laser was used at 755 nm wavelengths.

**Table 3:** Efficacy of tattoo removal and the effects on surrounding tissues of nanosecond and picosecond lasers according to wavelength.

Pulse duration	Wavelength (nm)	Efficacy	Surrounding tissue damage
**Nanosecond**	**532**	Red, Orange, Yellow, Green, Black	Thermal damage Vacuolation on dermis
	**1064**	Black	Extensive RBC extravasation Subcorneal separation
**Picosecond**	**532**	Red, Orange, Yellow, Green, Black	Least epidermal effect
	**755**	Green, Blue	Modest epidermal effect, but rapid healing
	**1064**	Black, some effect on other colors	Modest epidermal effect more prominent than following Pico-532 or Pico-755 treatment

The results demonstrate that the wavelength is more influential than pulse duration for the removal of each colored tattoo. Regardless of the pulse duration, the 532 nm laser was the most effective in clearing red, orange, and yellow colored tattoos and was mildly effective in removing green and black colored pigments. The overall effect and safety was better with the picosecond 532 nm laser. In removing black tattoos, the 1064 nm picosecond laser was the most effective. Another interesting point was that the 1064 nm picosecond laser demonstrated some effects on colored tattoos other than black ones, which can be presumed to be due to another mechanism with the wavelength besides the SPTL mechanism. The 755 nm picosecond laser exhibited excellent efficacy in green and, in particular, blue colored tattoos, but showed minimal effects on the other warm colors. When assessing the degree of damage to the surrounding tissues, the picosecond lasers, especially the 532 nm picosecond laser, tended to produce less tissue injury than did the nanosecond lasers. As a result, in comparison with the nanosecond laser, the picosecond laser demonstrated several advantages, including better tattoo removal ability and lesser side effects. These results are somewhat consistent with those of previous reports, in which picosecond laser pulses were found to be more efficient in clearing colored tattoos and produce less damage to the surrounding skin and scarring due to the low fluence level [4, 7–10].

The main mechanism of picosecond laser tattoo removal involves the fragmentation of the chromophore through both photothermal and photoacoustic effects. Picosecond lasers transmit light pulse lengths that are closer to the TRT of tattoo pigment molecules; therefore, they can deliver heat radiation more efficiently and can be destructively focused on the target area [5, 7, 11–13]. For example, the average size of carbon black in Indian ink is approximately 40 nm in diameter, while the TRT for 40 nm particles is approximately one nanosecond. The picosecond pulse can be thermally confined to the target because it is irradiated with a pulse duration of less than 1 nanosec [4, 14].

Additionally, in a fashion similar to the attainment of thermal confinement, it is possible to create a condition where all the pulse energy is deposited in the tissues before their volumes expand, thereby limiting the spread of the thermoelastic energy out of the target tissue. This condition is known as stress confinement and occurs when the pulse width is shorter than the characteristic acoustic relaxation time that is required for the heated sample to expand [11]. This photoacoustic laser-tissue interaction is known to be more suitable for reducing collateral damage than photothermal laser-tissue interactions, which are used in most nanosecond laser-based treatments. In our results, a unique pattern was frequently observed in the ultrastructural EM images of picosecond laser-treated samples regardless of the wavelength used. The "bubble-like" cavitation phenomenon appeared as a circular array of dark fine granules that seemed to surround the lysosomal lobes. In physics, this phenomenon has been reported to be caused by photoacoustic or photomechanical effects [14, 15]. This distinguishing ultrastructural feature suggests that the photoacoustic effect is another main mechanism of action in picosecond laser treatment.

As a result, the use of pulse widths between 10 and a few hundred picoseconds–shorter than both the thermal and acoustic stress relaxation times of pigment particles–maximizes energy confinement within the target volume [16]. For this reason, a picosecond laser can facilitate selective degradation into smaller sized ink particles and potentiate the efficacy of the treatment.

The ability to remove specific colors has been reported to be mainly due to the specific wavelength rather than the pulse duration [15]. However, we found that the 1064 nm picosecond laser exhibited overall moderate response to other colors as well as black. This is likely due to the photoacoustic effects of a very short pulse duration of 450 picoseconds, although further research is needed in this topic.

Since the present study is based on a single session of treatment in an animal model, there may be different results when these lasers are used in multiple sessions in humans. Despite this limitation, the strength of this study is that we quantified the differences between nanosecond and picosecond lasers at different wavelengths.

Picosecond lasers are still considerably more expensive and not more efficient than their nanosecond counterparts. Even though they have some limitations, picosecond lasers may demonstrate a greater ability to degrade smaller tattoo pigments through the photoacoustic effect. When the cost of picosecond lasers is reduced, they will be widely accepted as the optimal modality for tattoo removal.

## Supporting information

S1 Fig Clinical photographs of changes in orange, yellow, and blue pigments over time. The 755 nm picosecond laser was the most effective in removing blue colored tattoos, and the 532-nm wavelength laser was the most effective in removing orange and yellow colored tattoos at week 3. Picosecond lasers resulted in less epidermal damage post-treatment and rapid healing than the 532 nm nanosecond laser. This feature was observed notably in the process of the orange colored tattoo removal.
(TIF)

S2 Fig Ultrastructural analysis using electron microscope (EM) in the removal of blue (S2 Fig) colored tattoos. The EM results following treatment with picosecond lasers in all colors revealed a typical rimming pattern along the peripheries of lysosomal lobules. Unlike the picosecond laser, the nanosecond laser treatment resulted in tattoo particles that left the lysosomal structure and scattered into cellular spaces, thereby disrupting the cell structures.
(TIF)

S3 Fig Ultrastructural analysis using electron microscope (EM) in the removal of red (S3 Fig) colored tattoos. The EM results following treatment with picosecond lasers in all colors revealed a typical rimming pattern along the peripheries of lysosomal lobules. Unlike the picosecond laser, the nanosecond laser treatment resulted in tattoo particles that left the lysosomal structure and scattered into cellular spaces, thereby disrupting the cell structures.
(TIF)

S4 Fig Ultrastructural analysis using electron microscope (EM) in the removal of green colored tattoos. In all tattoo colors, EM findings demonstrated typical rimming patterns along the peripheries of lysosomal lobules after picosecond laser treatment.
(TIF)

S5 Fig Ultrastructural analysis using electron microscope (EM) in the removal of yellow colored tattoos. In all tattoo colors, EM findings demonstrated typical rimming patterns along the peripheries of lysosomal lobules after picosecond laser treatment.
(TIF)

S6 Fig Ultrastructural analysis using electron microscope (EM) in the removal of orange colored tattoos. In all tattoo colors, EM findings demonstrated typical rimming patterns along the peripheries of lysosomal lobules after picosecond laser treatment.
(TIF)

S7 Fig Hematoxylin & Eosin (H&E) staining of the biopsy specimens. Red and blue colored specimens were sampled right after the laser treatment. Dense RBC extravasation and dermal vacuolation were seen following 532 nm nanosecond laser treatment for red and blue pigments. These changes were less notable following treatment with the 755 nm picosecond laser and 1064 nm nanosecond laser.
(TIF)

S1 Table Components of the colored inks. (DOCX)
