# Supplementary figures and images for: Effects of picosecond laser on the multi-colored tattoo removal using Hartley guinea pig: A preliminary study
Source: PLoS One. 2018 Sep 6;13(9):e0203370. doi: 10.1371/journal.pone.0203370 (PMC6126847; doi:10.1371/journal.pone.0203370)

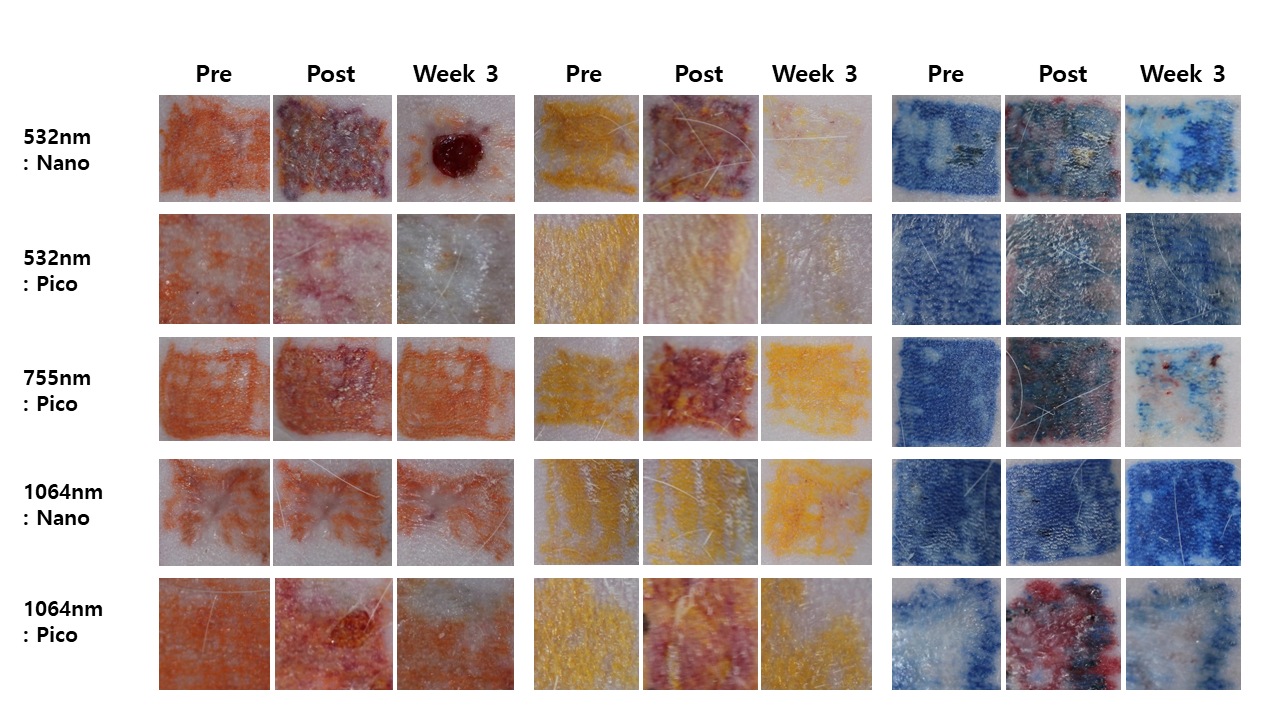

Supplement: S1 Fig — The 755 nm picosecond laser was the most effective in removing blue colored tattoos, and the 532-nm wavelength laser was the most effective in removing orange and yellow colored tattoos at week 3. Picosecond lasers resulted in less epidermal damage post-treatment and rapid healing than the 532 nm nanosecond laser. This feature was observed notably in the process of the orange colored tattoo removal. (TIF) [file pone.0203370.s001.TIF]

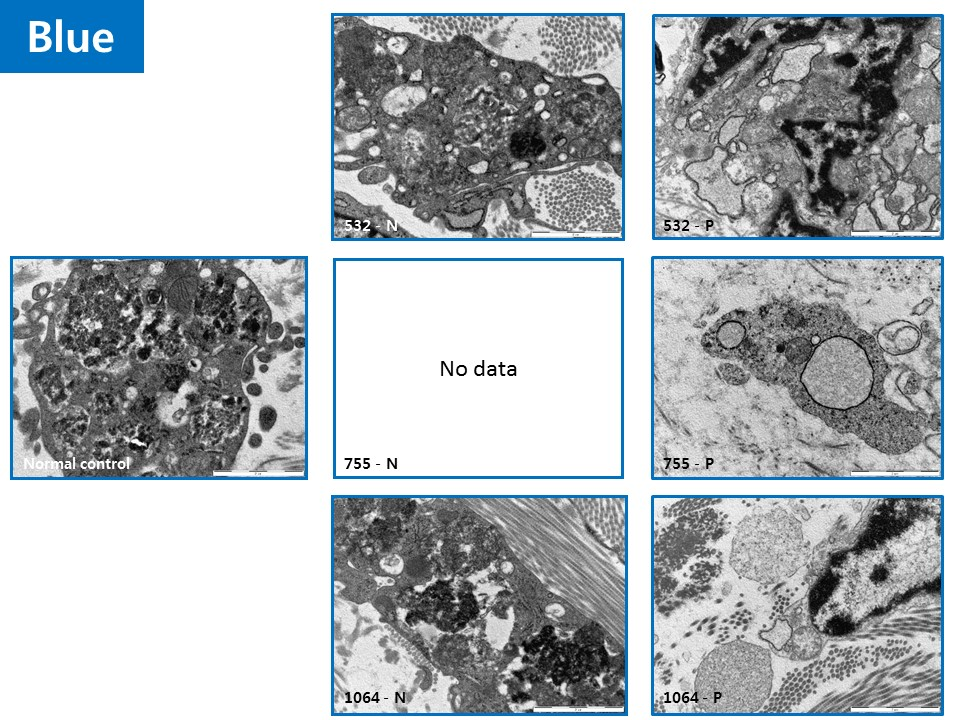

Supplement: S2 Fig — The EM results following treatment with picosecond lasers in all colors revealed a typical rimming pattern along the peripheries of lysosomal lobules. Unlike the picosecond laser, the nanosecond laser treatment resulted in tattoo particles that left the lysosomal structure and scattered into cellular spaces, thereby disrupting the cell structures. (TIF) [file pone.0203370.s002.tif]

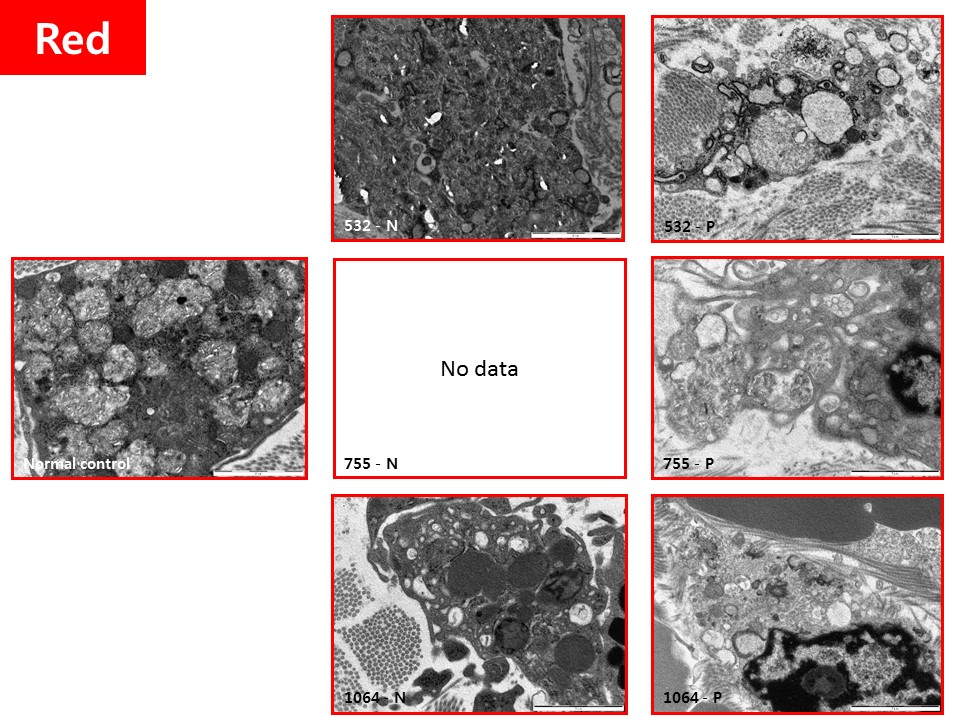

Supplement: S3 Fig — The EM results following treatment with picosecond lasers in all colors revealed a typical rimming pattern along the peripheries of lysosomal lobules. Unlike the picosecond laser, the nanosecond laser treatment resulted in tattoo particles that left the lysosomal structure and scattered into cellular spaces, thereby disrupting the cell structures. (TIF) [file pone.0203370.s003.tif]

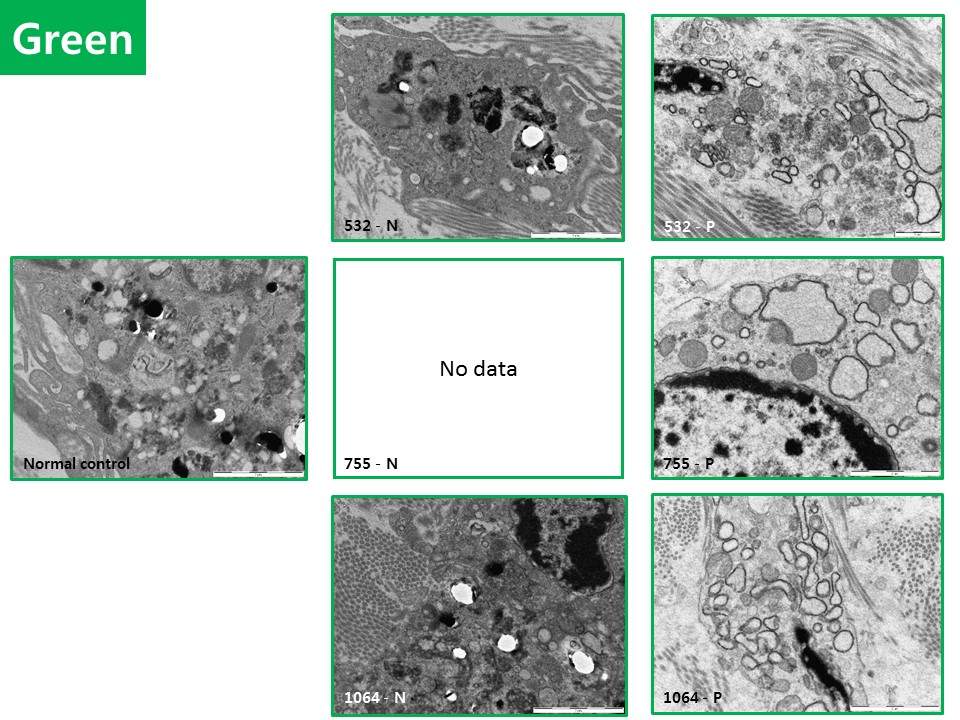

Supplement: S4 Fig — In all tattoo colors, EM findings demonstrated typical rimming patterns along the peripheries of lysosomal lobules after picosecond laser treatment. (TIF) [file pone.0203370.s004.tif]

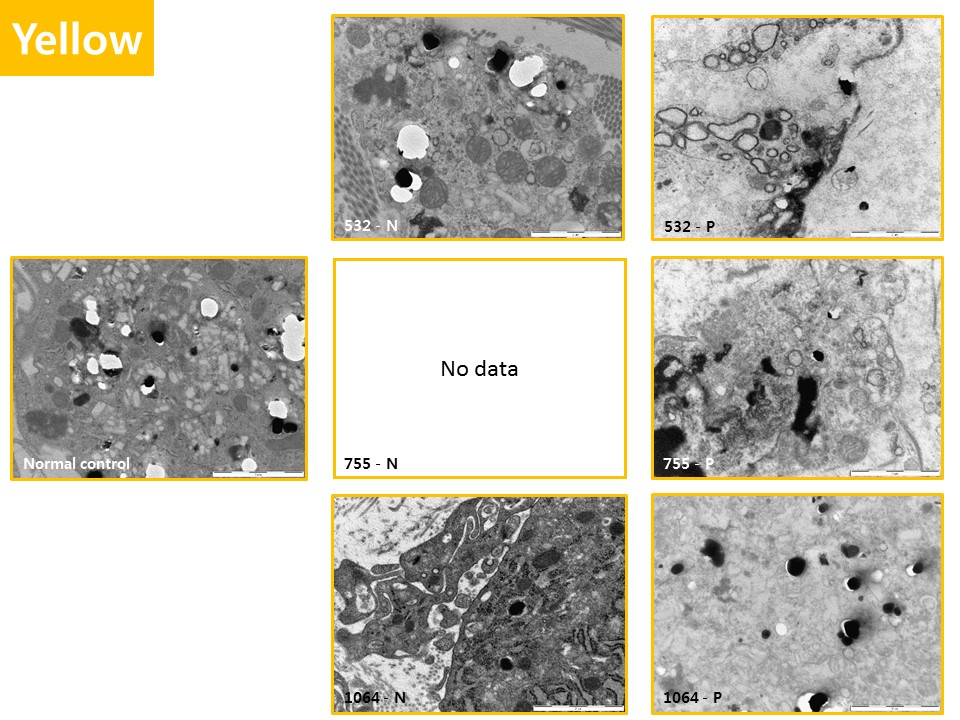

Supplement: S5 Fig — In all tattoo colors, EM findings demonstrated typical rimming patterns along the peripheries of lysosomal lobules after picosecond laser treatment. (TIF) [file pone.0203370.s005.tif]

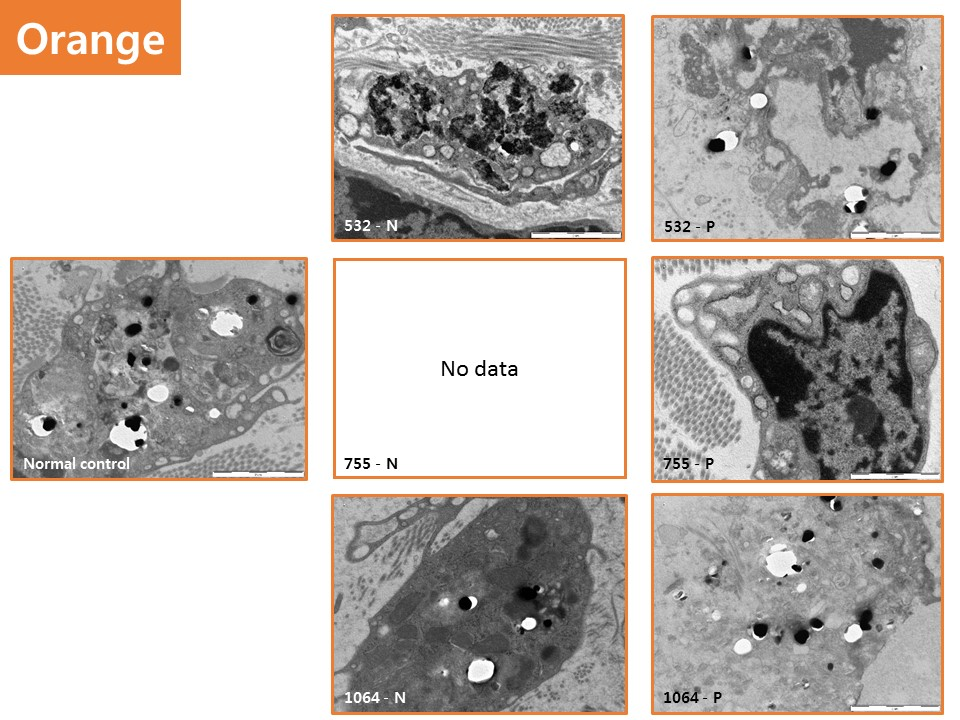

Supplement: S6 Fig — In all tattoo colors, EM findings demonstrated typical rimming patterns along the peripheries of lysosomal lobules after picosecond laser treatment. (TIF) [file pone.0203370.s006.tif]

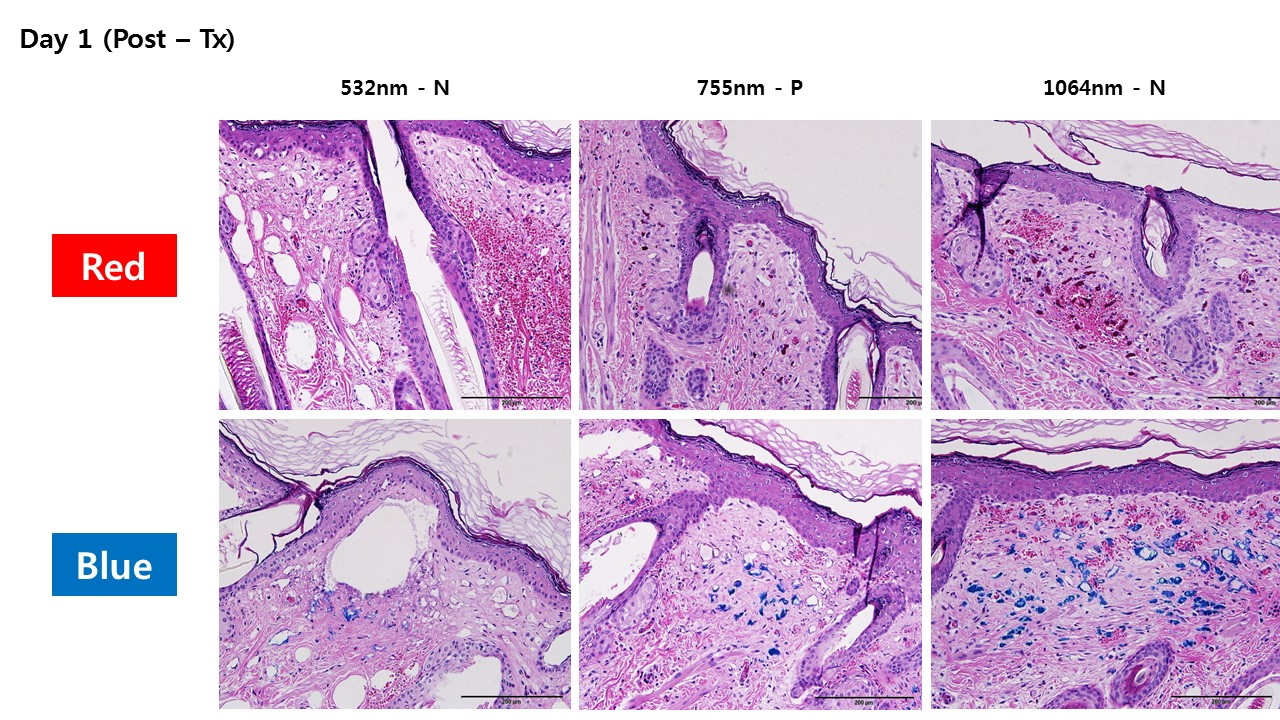

Supplement: S7 Fig — Red and blue colored specimens were sampled right after the laser treatment. Dense RBC extravasation and dermal vacuolation were seen following 532 nm nanosecond laser treatment for red and blue pigments. These changes were less notable following treatment with the 755 nm picosecond laser and 1064 nm nanosecond laser. (TIF) [file pone.0203370.s007.tif]
