# Supplementary material for: Effects of picosecond laser on the multi-colored tattoo removal using Hartley guinea pig: A preliminary study
Source: PLoS One. 2018 Sep 6;13(9):e0203370. doi: 10.1371/journal.pone.0203370 (PMC6126847; doi:10.1371/journal.pone.0203370)
Supplement: S1 Table — (DOCX) [file pone.0203370.s008.docx]

**Supplementary table 1. Components of color ink**

| **Color** | **Component** |
| --- | --- |
| Black | C.I.77266 : Carbon black |
|  | Distilled water |
|  | Glycerine |
|  | Isopropyl alcohol |
|  | Witch hazel |
| Blue | C.I.74160 : Copper phthalocyanine |
|  | Glycerine |
|  | Distilled water |
|  | Isopropyl alcohol |
|  | Witch hazel |
| Green | C.I.11740 : C_18_H_18_N_4_O_6_ (Organics), Pigment yellow 65 |
|  | C.I.77891 : Titanium dioxide (TiO_2_), Pigment white 6, titanium white |
|  | C.I.74160 : Copper phthalocyanine |
| Yellow | C.I.77891 : Titanium dioxide (TiO_2_), Pigment white 6, titanium white |
|  | C.I.11740 : C_18_H_18_N_4_O_6_ (Organics), Pigment yellow 65 |
|  | C.I.74160 : Copper phthalocyanine |
| Orange | C.I.11740 : C_18_H_18_N_4_O_6_ (Organics), Pigment yellow 65 |
|  | C.I.21110 : C_32_H_24_Cl_2_N_8_O_2_, Pigment orange 13 |
|  | C.I.77891 : Titanium dioxide (TiO_2_), Pigment white 6, titanium white |
| Red | C.I.12477 : C_25_H_20_N_4_O_4_, Pigment red 210 |
|  | C.I.11740 : C_18_H_18_N_4_O_6_ (Organics), Pigment yellow 65 |
|  | C.I.21110 : C_32_H_24_Cl_2_N_8_O_2_, Pigment orange 13 |
|  | Glycerine |
|  | Distilled water |
|  | Isopropyl alcohol |
|  | Witch hazel |
